# Sleep Health: An Opportunity for Public Health to Address Health Equity

**DOI:** 10.1146/annurev-publhealth-040119-094412

**Published:** 2020-01-03

**Authors:** Lauren Hale, Wendy Troxel, Daniel J. Buysse

**Affiliations:** 1 Program in Public Health; and Department of Family, Population, and Preventive Medicine; Renaissance School of Medicine, Stony Brook University, Stony Brook, New York 11794-8338, USA;; 2 Division of Behavior and Policy Sciences, RAND Corporation, Pittsburgh, Pennsylvania 15213, USA;; 3 Department of Psychiatry, School of Medicine, University of Pittsburgh, Pittsburgh, Pennsylvania 15213, USA;

**Keywords:** sleep, sleep health, public health, health disparities, health equity

## Abstract

The concept of sleep health provides a positive holistic framing of multiple sleep characteristics, including sleep duration, continuity, timing, alertness, and satisfaction. Sleep health promotion is an underrecognized public health opportunity with implications for a wide range of critical health outcomes, including cardiovascular disease, obesity, mental health, and neurodegenerative disease. Using a socioecological framework, we describe interacting domains of individual, social, and contextual influences on sleep health. To the extent that these determinants of sleep health are modifiable, sleep and public health researchers may benefit from taking a multilevel approach for addressing disparities in sleep health. For example, in addition to providing individual-level sleep behavioral recommendations, health promotion interventions need to occur at multiple contextual levels (e.g., family, schools, workplaces, media, and policy). Because sleep health, a key indicator of overall health, is unevenly distributed across the population, we consider improving sleep health a necessary step toward achieving health equity.

## INTRODUCTION

Poor sleep health is an underrecognized public health challenge strongly associated with morbidity and mortality (30). For example, more than 80% of older adults who report sleep disturbances describe at least one major mental or physical disorder, particularly depression, heart disease, pain, and memory problems (41). This strong overlap between sleep problems and other mental and physical health morbidities has led to a common misconception that sleep problems are merely an epiphenomenon of other conditions. However, over the past three decades, converging evidence demonstrates that sleep problems not only commonly co-occur with other morbidities, but also can play a causal role in the development of these conditions. This paradigmatic shift in viewing poor sleep health as a critical indicator and risk factor for health problems, rather than simply being secondary to those conditions, has important etiological and clinical implications for public health.

In this review, we describe current conceptualizations of sleep health and highlight findings linking sleep with cardiovascular disease (CVD), obesity, mental health, and neurodegenerative disease. Our review focuses on adults because most data address the health problems of midlife and older adults; understanding sleep health implications in childhood remains a pressing research priority. We also discuss the socioecological model of sleep health and the potentially mediating role of sleep in understanding health disparities. We conclude with recommendations and opportunities for interventions, future research areas, and the importance of promoting sleep health for achieving health equity. This review should not be considered exhaustive, but rather a summary of key, contemporary findings that, in our opinion, represent the state of the science on the role of sleep in public health and promising future directions.

## SLEEP HEALTH: IT IS MORE THAN THE SUM OF ITS PARTS

In prior decades of sleep research, scholars tended to focus on a narrow range of sleep problems, which were disease oriented rather than positively framed as health oriented (17). The most common sleep characteristics studied were short and long sleep duration; the most common symptoms studied were insomnia symptoms (e.g., trouble falling asleep, staying asleep, and waking up too early), and the most common disorder studied was sleep apnea.

Epidemiologic data on each of these sleep indicators present an alarming picture. For example, current consensus panel recommendations indicate that adults should sleep a minimum of 7 h per night (15, 36), yet the Centers for Disease Control and Prevention (CDC) reports that about 35% do not meet that recommendation (20). Insomnia symptoms refer to patient-reported difficulty falling asleep or staying asleep or awakening too early. Insomnia disorder refers to insomnia symptoms that persist for some duration (typically 1–3 months) and that cause significant distress or impaired waking function. Worldwide between 30% and 35% of adults suffer from insomnia symptoms, and about 10% of the population meet diagnostic criteria for insomnia disorder (93). Obstructive sleep apnea (OSA), another prevalent sleep disorder with public health implications, is defined by recurrent episodes of not breathing (apnea) or reduced airflow (hypopnea) during sleep, despite continued respiratory effort. OSA leads to intermittent hypoxemia and repeated arousals, which can compromise cardiovascular function. Prevalence estimates for OSA vary widely, between 3% and 10% of the general population; however, OSA is likely severely underdiagnosed, particularly among African Americans, overweight individuals, and older adults (42, 69). These statistics highlight the multidimensional nature of sleep and suggest that poor sleep characteristics, sleep symptoms, and sleep disorders are common and costly to public health.

The emerging concept of “sleep health” presents a more holistic view of sleep, including multiple domains of sleep characteristics, including regularity, alertness, timing, efficiency, and satisfaction, rather than individual symptoms and disorders (17). Unlike sleep symptoms and disorders, multidimensional sleep health can be measured as a continuous variable to characterize every individual in the population. The concept of sleep health provides a useful heuristic for integrating existing research, identifying gaps in the literature, and recognizing promising future directions for research and clinical intervention. Furthermore, this broader conceptualization of sleep as a multidimensional entity of health coheres with the World Health Organization’s view, which describes “health” in general, as a “state of complete physical, mental and social well-being and not merely the absence of disease or infirmity” (140). Multidimensional sleep health recognizes that sleep occurs at the individual level and also in a larger socioecological context. This perspective is consistent with two key developments in sleep science over the past two decades: the recognition of substantial inequities in sleep that parallel other racial/ethnic and socioeconomic disparities in other health outcomes (53, 54, 67, 102), and the recognition that determinants of sleep health go beyond the individual, encompassing socioenvironmental influences as well (55, 66). Thus, sleep health not only serves as a key indicator of overall health and equity, but also provides an opportunity for multiple levels of intervention (66). Although sleep health and its components are associated with a broad range of morbidities, we next highlight examples from the recent literature on the association between sleep and key health outcomes, including CVD, obesity, mental health, and neurogenerative diseases.

## SLEEP HEALTH AS A KEY HEALTH INDICATOR

### Cardiovascular Disease

CVD is the leading cause of death globally (141). While sleep disturbances are common among individuals with CVD (85), a burgeoning evidence base demonstrates the role of sleep characteristics and disorders in contributing to CVD morbidity and mortality (57).

Most of the existing epidemiologic evidence on sleep and CVD risk has focused on isolated sleep characteristics, primarily sleep duration, or specific sleep disorders, primarily OSA. For example, a 2011 meta-analysis of 15 studies, involving more than 400,000 individuals, showed that short sleep duration (<7 h) is associated with incident coronary heart disease (CHD) and stroke, as well as increased risk of CHD mortality (20). Long sleep duration (>9 h) is associated with increased risk of incident CHD, stroke, and total CVD events (20). While much of this research comes from studies of single-item, self-reported assessments of habitual sleep duration (20), these findings are supported by studies that objectively measured (i.e., via actigraphy or polysomnography) sleep characteristics (7, 62, 133). Other sleep characteristics including sleep quality, regularity, and timing of sleep have been associated with CVD risk factors (62, 80). For example, Huang & Redline (62) found that greater objectively measured variability in sleep timing and duration was associated with higher prevalence and incidence of metabolic dysregulation, independent of sleep duration and other lifestyle risk factors.

Beyond these isolated sleep characteristics, research has also examined links between clinical sleep disorders (e.g., OSA and insomnia) and CVD risk and events. A meta-analysis of prospective cohort studies indicates that moderate-to-severe OSA predicts increased CVD risk, particularly stroke risk (36). Insomnia has been examined in relation to CVD both as a symptom and as a discrete disorder. Although insomnia is the most common sleep disorder and insomnia symptoms are highly prevalent in the general population, relatively less research has focused on insomnia as a risk factor for the development of CVD risk, compared with OSA. The available evidence is somewhat equivocal, perhaps owing to heterogeneity in the correlates and consequences of insomnia symptoms versus disorder and because certain subtypes or combinations of symptoms may be more strongly linked with CVD risk than others (68). For example, Troxel and colleagues (125) found that specific symptoms of insomnia, including difficulty falling asleep and poor-quality sleep, but not a syndromal definition of insomnia, were significant predictors of the development of metabolic syndrome in a cohort of black and white men and women. Furthermore, in a series of landmark studies, Vgontzas and colleagues (40, 131, 132, 134) demonstrated that insomnia coupled with short polysomnographic sleep duration strongly and significantly predict hypertension, metabolic dysfunction, depression, and mortality relative to insomnia with normal sleep duration. Thus, the insomnia short sleep phenotype may specifically convey higher risk for cardiovascular outcomes.

Collectively, these findings demonstrate the importance of considering multiple indicators of sleep health simultaneously as they relate to CVD and the underlying mechanisms that may explain why some sleep symptoms or disorders are more strongly linked with CVD outcomes. For example, laboratory-based studies provide convincing evidence that short sleep duration may contribute to CVD risk, in part through disruptions in metabolic dysregulation, autonomic functioning, and inflammatory processes (125). Similarly, insomnia is conceptualized as a disorder of physiologic and emotional hyperarousal, which may be associated with heightened sympathetic nervous system activation, hypercortisolemia, and activation of nuclear factor (NF)-κB [which serves a critical role in cellular inflammatory signaling (65)], all of which are implicated in the pathogenesis of CVD.

Building on the robust evidence linking sleep symptoms and disorders with CVD risk, intervention studies would provide the strongest demonstration of sleep as a causal risk factor. Such intervention-focused research (51, 114), however, has lagged far behind the observational studies of sleep and CVD risk, and the limited data to date are inconclusive. For example, a large, randomized clinical trial found that CPAP (continuous positive airway pressure) treatment did not prevent CVD events among patients with prevalent CVD (92). However, these findings have several limitations, including the fact that patients in this trial generally presented with OSA without daytime sleepiness and that CPAP compliance was low (3.3 h per night, on average). Future research should explore the hypothesis that specific OSA phenotypes, particularly those who present with more severe OSA and those with daytime sleepiness, may derive the greatest cardiovascular benefits from CPAP treatment (44). Furthermore, behavioral strategies to support the uptake of and compliance with treatments for OSA require further investigation (32). Finally, short-term studies focused on interventions to extend sleep duration and/or improve sleep quality suggest that improving sleep health holds promise as a strategy to reduce cardiovascular risk (51).

### Obesity

The obesity epidemic has multiple determinants ranging from genetics to eating behavior to environmental forces (33). Recent research on sleep health and circadian science highlights the role of sleep as an additional contributing factor to weight gain and obesity (6, 33, 86). Cross-sectional data indicate that short sleep duration is associated with increased odds of obesity among both children and adults. For example, a 2008 meta-analysis of data from 18 adult studies (*n* > 600,000) indicates that short sleep duration is associated with a 55% increase in odds for obesity (21). Although most of this research has relied on cross-sectional data, a meta-analysis of 11 studies (*n* ~ 200,000) showed that the risk of incident obesity is 45% higher among those who report short sleep duration, with no longitudinal association between long sleep duration and incident obesity (144). Other dimensions of sleep health, including sleep variability, sleep timing, daytime napping, and low sleep efficiency, have all been associated with increased obesity, but they have been studied less frequently than sleep duration (96). There is less evidence of an association between insomnia disorder and obesity (25, 39). A meta-analysis of 67 studies showed no association between insomnia disorder and obesity and only a small positive association between insomnia symptoms and obesity (25).

Sleep health may influence obesity through multiple pathways, including the possibility that people who sleep less have more time available to eat per day (75, 115, 118). Experimental data show that sleep deprivation changes the hormones that regulate appetite, including increases in the hormone ghrelin, which causes hunger, and decreases in the hormone leptin, which regulates fullness (116, 122). In addition, sleep restriction causes behavioral changes such as larger portion size and food choices with higher caloric intake (19, 61). Finally, sleep loss also affects glucose metabolism (75, 115).

The circadian timing system may also serve as a critical link between sleep and metabolism. Circadian timing both influences and is influenced by the metabolic state of cells and organisms. Cellular energy metabolism at the molecular level is linked directly to the molecular circadian clock (88, 89). At the behavioral level, food can entrain the circadian timing system, and eating at an inappropriate circadian phase promotes weight gain and obesity in both animals and humans (6, 9, 88). Thus, restricting feeding/eating times within the appropriate circadian phase can both prevent obesity and result in weight loss (47, 59).

While there is strong and consistent evidence for the link between sleep health and obesity, it is essential to tease out the potential roles of reverse causality, bidirectionality, and third factors. Indeed, obesity (and related lifestyle factors such as diet and physical activity) may contribute to poor sleep health and sleep disorders, especially OSA (110). Despite these concerns, sleep and circadian-focused interventions, including sleep extension, regularizing sleep schedules, appropriate meal timing, and time-restricted eating, may serve as potential strategies for promoting weight loss, weight management, and improved glucose metabolism (28, 81, 123).

### Depression and Mental Disorders

Sleep problems are a symptom of virtually every mental health condition, and sleep problems are even more prevalent in individuals with mental health problems as compared with the general population (78). For example, 65–90% of adults with major depressive disorder (MDD) report sleep problems, and 90% of children with depression report disturbed sleep (78). Sleep problems have traditionally been viewed as a consequence or symptom of a mental health condition rather than as a specific etiological agent that may play a causal role in the development of mental health issues. However, owing to robust, longitudinal, and prospective research, there is emerging consensus that sleep problems are a cause as well as a consequence of mental health problems. In fact, in 2005 the National Institutes of Health (NIH) issued a statement recommending that sleep problems no longer be routinely conceptualized as secondary to another disorder (95). This shift in recognizing the bidirectional associations between sleep and mental health has substantial implications for understanding the etiology of mental health.

The strongest evidence to date concerning a causal role of sleep in the development of mental health problems comes from prospective studies of sleep problems, particularly insomnia, and depression. For example, a meta-analysis of 34 cohort studies involving more than 170,000 participants with an average follow-up period of 60.4 months showed that insomnia symptoms significantly predicted the subsequent development of MDD (82). The pooled estimate across studies showed that those with insomnia at baseline had a more than twofold increased risk of developing MDD. Others have demonstrated prospective associations between insomnia and suicide (143) and anxiety (94, 103). More recent evidence has examined the role of insomnia in predicting the development of other mental health disorders including post-traumatic stress disorder (PTSD), bipolar disorder, and psychosis. For example, sleep problems and PTSD are highly prevalent in military populations and may be further heightened by prolonged and repeated deployments, as seen in the US military (124). Limited evidence suggests that insomnia predicts the development of PTSD in service members postdeployment (46, 138). Furthermore, sleep loss can precipitate mania symptoms in the context of bipolar disorder (58), and recent evidence indicates that sleep problems and disorders may increase the risk of psychosis and severity of psychotic episodes (84, 107).

The growing recognition that sleep problems not only co-occur with mental health problems but can also predict the onset of mental health problems has significant treatment implications and highlights important opportunities to identify transdiagnostic mechanisms that contribute to both poor sleep health and poor mental health. For example, untreated sleep problems predict poorer treatment prognosis (127) and greater likelihood of relapse (35), even for the most effective pharmacologic and/or behavioral treatments for depression. Moreover, recent findings from a large, randomized clinical trial demonstrated that treatment of insomnia, with cognitive behavioral therapy, led to reductions in psychotic experiences among university students (43). These promising findings provide strong evidence for a causal role of sleep disturbances in the development and exacerbation of mental health disorders and the potential for treating sleep problems as an important intervention target in the armament of strategies to support mental health.

## NEURODEGENERATIVE DISEASES

Sleep is related to neurodegenerative disorders in three fundamental ways: First, patients with such disorders frequently have sleep problems and sleep disorders; second, sleep problems and sleep disorders may be risk factors for the subsequent development of neurodegenerative disorders; and finally, sleep may relate to the pathophysiology of these conditions. Although sleep has been examined in several neurodegenerative conditions, we focus on the two most common, Alzheimer’s disease and Parkinson’s disease.

### Alzheimer’s Disease

The fundamental pathophysiology of Alzheimer’s disease involves deposition of extracellular plaques containing amyloid-β (Aβ) and intracellular neurofibrillary tangles consisting of accumulated tau proteins throughout the brain. Structural neuroimaging studies typically show a loss of cortical and hippocampal volume, and positron emission tomography shows regional hypometabolism, particularly in the medial frontal, cingulate, entorhinal, and temporal cortices, and hippocampus (91). The clinical features of Alzheimer’s disease include memory impairment, particularly short-term memory, coupled with impairments in complex attention, executive function, language, and social cognition (4). Incidence and prevalence of Alzheimer’s disease increase with age, with prevalence of 11% at age 65 increasing to 32% by age 85 (60).

Up to 45% of individuals with Alzheimer’s disease have sleep-wake disturbances, and sleep problems track with disease severity (90, 135). Disturbed sleep complicates caregiving and is a risk factor for nursing home placement (105). The most common sleep disturbance is frequent awakenings at night, sometimes accompanied by behavioral agitation as part of sundowning syndrome. Conversely, Alzheimer’s disease is associated with increased daytime napping. The combined effects of nighttime and daytime sleep disturbance suggest reduced amplitude of diurnal or circadian rhythms (5), which is consistent with the loss of retinal ganglion cells and suprachiasmatic nucleus neurons (79, 120, 121). Alzheimer’s disease is also associated with progressive changes in specific sleep stages, including progressive reduction in REM (rapid eye movement) sleep and deep NREM (non-REM) sleep (slow-wave sleep) and loss of typical NREM EEG (electroencephalography) features, including sleep spindles and K complexes (101). Because sleep deprivation, sleep fragmentation, and reduction in slow-wave sleep are associated with cognitive function in healthy individuals, it is reasonable to hypothesize that such sleep disturbances could worsen cognitive function in patients with Alzheimer’s disease.

Patients with dementia are at an increased risk for sleep disorders, including OSA relative to age-matched controls, with prevalence estimates of OSA as high as 50–70%; this risk is elevated among individuals who are carriers of the APOE-ε4 (apolipoprotein-E) allele (11, 38). Both OSA and insomnia increase the risk for subsequently developing Alzheimer’s disease (99, 146). Moreover, the sleep and breathing disturbances of insomnia and OSA may compound synaptic dysfunction and cognitive impairment in patients with dementia (87).

Recent studies both in animal models and in humans have suggested a plausible link between Alzheimer’s disease pathology and sleep. Neurotoxic Aβ and tau proteins are cleared from brain tissue via the glymphatic system, a perivascular network that circulates interstitial fluid (12). Glymphatic clearance is markedly higher during sleep, and particularly slow-wave sleep, relative to wakefulness (145). Disrupted sleep and reduced slow-wave sleep may result in reduced glymphatic clearance of Aβ and tau proteins and their accumulation in brain tissue; these proteins, in turn, interfere with normal synaptic function, which could further impair sleep (87). Supporting this hypothesis, poor subjective and objective sleep quality are associated with increased amyloid in brain tissue and decreased amyloid in cerebrospinal fluid (72, 117).

### Parkinson’s Disease

Parkinson’s disease (PD) is a chronic degenerative neurologic disorder characterized by neuronal cell loss in the substantia nigra, a brain stem nucleus that is a major source of dopamine. The subsequent reduction in brain dopamine accounts for the cardinal symptoms of PD: resting tremor, slowed movement, and rigidity. However, PD involves more wide-ranging symptoms, including sleep disturbances and disorders, cognitive impairment, depression, and autonomic instability. PD is associated neuropathologically with the aggregation of α-synuclein protein fibrils into Lewy bodies, which are deposited throughout the brain stem and cortex. Lewy bodies are also observed in other degenerative neurologic disorders, including dementia with Lewy bodies and multiple system atrophy, which, together with PD, are sometimes referred to as α-synucleinopathies. PD has a prevalence of 0.1–0.2% in the overall population, and 1% in adults over age 60 (129).

Sleep symptoms and disorders are common among individuals with PD (24). About 33% of PD patients have clinically significant insomnia, most often with sleep maintenance difficulties; objective indicators of shorter and more interrupted sleep are also common (147, 148). Excessive daytime sleepiness (EDS) is observed in 30–50% of patients with PD, with increasing prevalence as the disease progresses (24). Some data suggest that EDS may be a prodromal symptom or risk factor for developing PD (1). Multiple factors may contribute to both insomnia and EDS in PD, including medications, other sleep disorders, and comorbid psychiatric conditions; likewise, both insomnia and EDS may contribute to impaired quality of life and function as well as cognitive and motor symptoms (24).

Although sleep disorders such as restless legs syndrome (RLS) and OSA can be observed in patients with PD, their prevalence is no higher in this disorder compared with the general population (24). On the other hand, REM sleep behavior disorder (RBD) bears a specific relationship to PD and other synucleinopathies. RBD is characterized by a loss of the usual muscle atonia that accompanies REM sleep. As a result, patients may act out their dreams, which often include violent content, posing risk of physical injury to self and bed partners. RBD occurs in 30–50% of patients with PD (104, 111), but more notably, it may precede the motor manifestations of PD and other α-synucleinopathies by many years. Thus, RBD is considered a prodrome for PD, with cumulative risk of PD of up to 90% when patients are followed for up to 15 years (63). The Braak hypothesis of PD suggests that neuronal involvement begins in the brain stem and gradually ascends to sub-cortical and cortical regions (15). The appearance of RBD early in the course of PD is consistent with this hypothesis because it appears to result from brain stem nuclei dysfunction (13, 45).

## EMBRACING A SOCIOECOLOGICAL MODEL OF SLEEP HEALTH

Having reviewed sleep health as an indicator of health outcomes, we shift our attention to focus on sleep health as a contributor to health disparities, as well as the role of upstream social and contextual factors that shape sleep health (49, 66) and opportunities for informing interventions and policy.

Widely observed differences in sleep health across the population may have implications for explaining widely observed gaps in health, by race, socioeconomic status, and other social dimensions (66). For example, a recent study found that 41% and 58% of racial differences in cardiometabolic risk (as measured by 7 biomarkers) were accounted for by differences in sleep duration and sleep efficiency, respectively (31). Thus, a growing body of research suggests that sleep may serve as a mediating factor on the pathway toward broader health disparities. Efforts to address differences in sleep across the population may have the added benefit of reducing other disparities in health and well-being (66). Furthermore, the recognition that sleep health is socially patterned has given rise to a more comprehensive examination of the multilevel determinants of sleep health and sleep health disparities.

**Figure 1:**
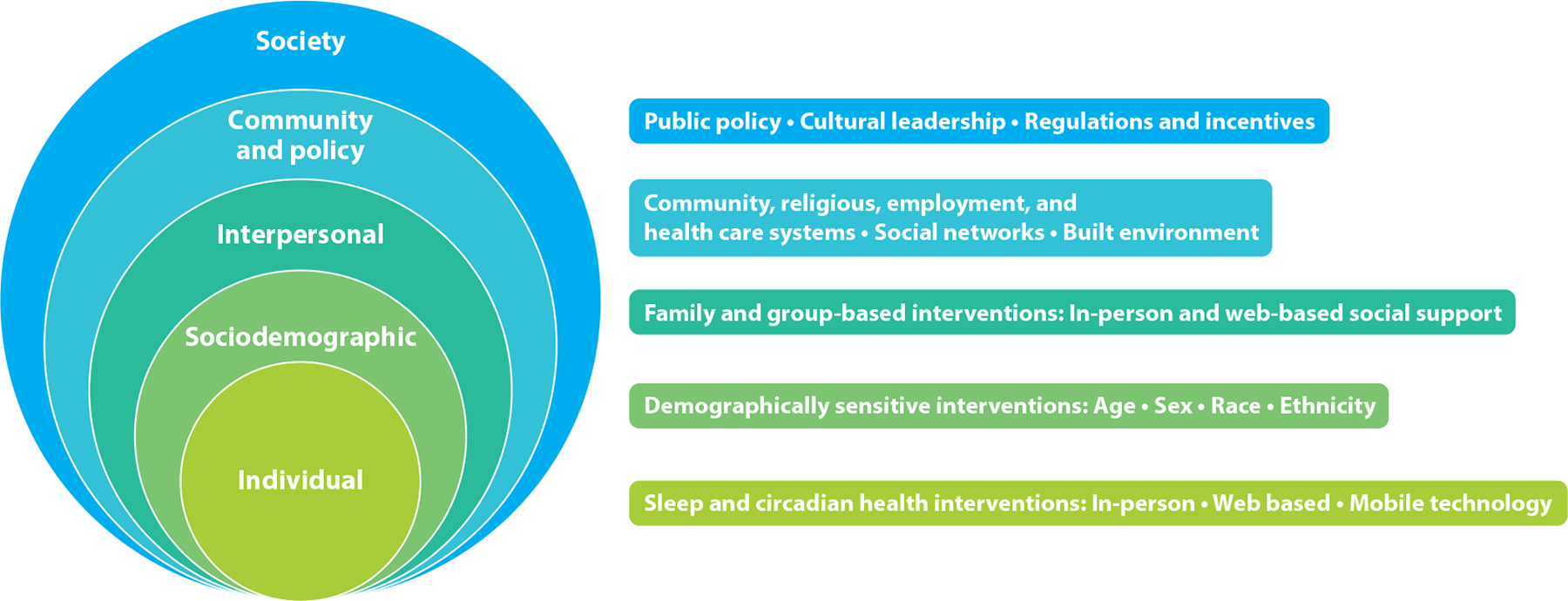
The socioecological model of sleep and circadian health with corresponding multilevel intervention strategies.

Socioecological models provide a framework for thinking about the dynamic interplay among individual, family, and contextual factors (119). As depicted in Figure 1, sleep health is a function of multiple levels of influence, ranging from individual behaviors to interpersonal factors, community influences, and broader societal influences (66). Each of these levels of influence and examples of relevant interventions are briefly summarized in turn.

### Individual Behavioral Factors

Individual behavioral factors, such as sleep habits and behaviors, have typically been targeted as the primary determinants of individual sleep health (55, 64). Standard recommendations for healthy sleep behaviors include maintaining regular bedtimes and wake times across the week; limiting caffeine, alcohol, and other substances; creating a cool, dark, and quiet bedroom; restricting digital media in the hour before bedtime; and allowing adequate opportunity for sleep (64). With the pervasive use of mobile digital technology, exposure to media content and melatonin-suppressing light from screens before bedtime may be particularly disruptive to sleep onset, quality, and duration (22, 26). Opportunities for intervention at the behavioral level should include sleep and circadian health interventions, in-person and Web-based coaching, and use of mobile technology.

Unfortunately, there is limited empirical support for “sleep hygiene” recommendations as stand-alone interventions when offered without personalization (64). Therefore, encouraging better sleep behaviors at the individual level alone may not be enough to improve sleep health (55).

### Sociodemographic Factors

Sleep health is socially patterned by a range of less mutable sociodemographic characteristics (e.g., race/ethnicity, education). For example, studies from childhood (50, 113) through adulthood (102, 109) consistently find shorter sleep durations and often poorer sleep health among blacks compared with whites. Other individual-level socioeconomic factors, including higher levels of education and marriage, are generally positively associated with better sleep health outcomes (53, 77). While some of these associations may not be causal, they point to the possibility of higher levels of stress due to everyday challenges encountered by more disadvantaged populations, owing to financial insecurity and less autonomy over their life choices (52, 55). Awareness of sociodemographic differences in sleep health can lead to the development and implementation of culturally and demographically sensitive interventions, as well as targeted provision of services to higher-risk populations.

### Interpersonal Factors

At the interpersonal level, sleep health is linked to a wide range of social processes (48). Being in supportive relationships is associated with less troubled sleep, whereas relationship stress is associated with more troubled sleep (3, 128). High-quality relationships may provide a sense of safety, reduce vigilance, promote positive sleep-related behaviors, and reduce physiologic responses that interfere with sleep (128). In contrast, loneliness and social isolation are associated with poorer sleep health indicators (10, 18). Outside of one’s romantic and close social relationships, other forms of interpersonal interactions, including workplace interactions or other common social experiences, may affect sleep health and contribute to sleep health disparities. For example, a systematic literature review identified 17 studies demonstrating an association between higher levels of discrimination and poorer sleep (112). At the interpersonal level, family and group-based interventions may be an effective strategy, especially for pediatric sleep health, to help all members of a household recognize that their behaviors may contribute to the bedtime and sleep patterns of others.

### Community Factors

At the community level, environmental characteristics (e.g., physical conditions and social environment) (70, 71, 73, 126) may have direct or indirect effects on sleep. Physical conditions refer to aspects of the physical (e.g., buildings, roads, traffic patterns, trees) and ambient environment (e.g., noise, temperature, light pollution) that can affect sleep characteristics (29, 73, 76). For example, inopportune light exposure from streets and commercial buildings may suppress melatonin, delay sleep onset, prolong sleep latency, and shorten total sleep time (29, 76, 97). Neighborhood social environment refers to factors such as social cohesion, safety, exposure to crime, and socioeconomic advantage, all of which are associated with sleep health (71, 126), ostensibly by influencing psychological and physiological stress responses. The community level also includes employment characteristics (e.g., number of hours worked, workplace culture, timing of work/school), which can be regulated at the local level or influenced by policy (8, 16, 98). For example, early school start times, which are in conflict with the known biological delay in adolescents’ sleep-wake patterns, are a key community-level factor that contributes to short sleep duration among adolescents (2, 139). Finally, interactions with the health care system—including access to care, costs, and the actual physical environment of sleeping inside a hospital—can affect sleep health (106). Interventions at the community level include opportunities to adjust workplace environments, school start times, health care systems, and features of the built and social environment to improve sleep health.

### Societal Factors

At the broadest level, we refer to societal factors such as local, state, and federal policies that can affect sleep health. Social and health care policies affect financial stress, safety concerns, residential segregation practices, and individual autonomy, which may restrict or facilitate opportunities for achieving good sleep health (52). At the societal level, interventions take the form of cultural leadership (e.g., public health priorities and sleep health awareness campaigns) and public policy, including regulations and incentives (e.g., regulation of shiftwork, elimination of daylight saving time, tax incentives for corporate wellness programs) (8, 108).

## IMPLICATIONS FOR POLICY AND INTERVENTIONS

In summary, we have presented two arguments: First, sleep health is critically important and underrecognized as a correlate, and likely causal factor, in overall health and specific health conditions. Second, social and environmental determinants of sleep health are often outside the scope of individual behavior, presenting opportunities for multilevel interventions to improve sleep health and reduce disparities. The notion that sleep health is modifiable through social and environmental changes is key to thinking about much needed intervention research. The field is shifting to recognize that since sleep health affects everyone, it belongs in the domain of public health. As such, multilevel preventive strategies are needed to improve sleep health (8).

One notable example is the policy of delaying high school start times to the American Academy of Pediatrics recommended time of 8:30 am or later (2). School districts that have shifted their start times have demonstrated sleep and health benefits that include longer sleep duration, less sleepiness, less tardiness, better academic outcomes, and fewer motor vehicle accidents (14, 139). Despite the compelling science and consensus statements by numerous medical organizations supporting later high school start times (2, 139), fewer than 20% of middle and high schools in the United States adhere to recommendations to start school no earlier than 8:30 am (23). However, in October 2019, California became the first state to pass legislation mandating that middle schools start no earlier than 8 am and high schools no earlier than 8:30 am. The sleep research community has an opportunity to collaborate with public health and communication scholars to improve communication and dissemination strategies to mitigate concerns (56).

Other broader-scale public health sleep interventions (e.g., sleep health education awareness campaigns, workplace policies, etc.) implemented in coordination with relevant stakeholders hold potential to improve sleep health at the population level. Individual-level interventions have focused primarily on sleep disorders—and while some, like cognitive behavioral therapy for insomnia, are very effective, they are underutilized and have limited population-level impact. It is also critical that interventions focus on scalability and issues of access, and cultural sensitivity, to ensure that populations most vulnerable to poor sleep health are included (142).

## PRIORITIES FOR FUTURE RESEARCH

Research on sleep health has grown rapidly in the last decade. With this new research come challenges and opportunities. As part of our overview of the state of the science, we highlight some priorities for future sleep health research.

### Address Methodological Concerns

One of the enduring challenges of studying sleep health is determining how to appropriately measure the construct to optimize the competing tensions of accuracy and scalability. Several research groups are exploring novel strategies for incorporating multiple dimensions of sleep health in population-based studies (74, 136). Another approach seeks to incorporate variability in sleep across the 24 h of a day and over the week into a single measure (83). Furthermore, it is difficult to study sleep owing to its bidirectional relationships with so many concurrent variables (e.g., diet, physical activity, stress). Longitudinal within-person analyses in which sleep health is treated as both a predictor and an outcome on a daily scale can help address this concern more effectively. In addition, exploratory data analysis methods, such as machine learning, may identify directions for future experimental studies (136, 137). Methodological advances in sleep health research will help underscore the temporal dynamics between various dimensions of sleep health and everyday life.

### Study Vulnerable Populations

In light of the potential importance of sleep health for reducing disparities in health (31), the most vulnerable populations in terms of poor sleep health should be considered a top research priority. Some of these populations who are at risk for poor sleep health, including racial/ethnic minorities and adolescents, have been investigated in numerous studies mentioned above. However, there are many other subpopulations that remain understudied by the sleep research community, such as prison inmates (34), those living in homeless shelters (27), American Indian/Alaska Native populations (37), and patients in hospitals and nursing facilities (106, 130).

### Improve Implementation Science

A major challenge for future research is understanding how to translate the science behind sleep into policy and interventions that are implementable, effective, and sustainable. The NIH and the Sleep Research Society (SRS) conducted a joint workshop during which they created recommendations for addressing the implementation gap, in which the scientific knowledge regarding sleep health is not yet translated into preventive interventions or health care treatments (100). The authors encourage the use of multilevel strategies in which sleep health is addressed at the individual, provider, community, health system, and policy levels (100). Other efforts to address sleep health as a public health issue include the National Healthy Sleep Awareness Project (CDC, American Academy of Sleep Medicine, SRS), Healthy People 2020 (CDC), and various consensus panels (SRS, National Sleep Foundation). These collaborative attempts to address this implementation gap show promise for future research on improving population sleep health.

## CONCLUSIONS

The conceptual transition from focusing on sleep duration and sleep disorders to the positive notion of sleep health provides a more holistic health-oriented perspective on sleep. Furthermore, we contend that sleep health is a public health opportunity that has been underrecognized by both the sleep research and public health communities until recently. Indeed, the current state of the science, some of which is summarized above, indicates that the benefits of sleep health to the population affect a wide range of critical health outcomes.

Conversely, poor sleep health aligns with social disadvantage, including in racial/ethnic minorities, the homeless, and institutionalized populations. Emerging evidence suggests that the uneven distribution of sleep health across the population contributes to health inequities. The mission of the American Public Health Association is “to improve the health of the public and achieve equity in health status” (https://www.apha.org/about-apha/our-mission). To the extent that sleep health is modifiable through changes in the socioecological environment, and sleep health is critical for health status, we consider improving sleep health a necessary step toward achieving health equity.

Toward that end, the final take-home point is that, just as we need to take a more holistic approach to considering how we define sleep health, we also need to take a more holistic approach to considering multilevel social and environmental determinants of sleep, consistent with a socioecological model. As with other public health epidemics, such as obesity, that have increasingly recognized the limitations of individual-level interventions alone, sleep health and public health researchers will benefit from adopting a multilevel approach for developing and disseminating evidence-based and scalable interventions. Sleep health promotion efforts should be considered at all levels of the socioecological model from the individual level up through the societal level. Employers, teachers, community members, health care providers, the media, and policy makers all have a role to play in changing and promoting a culture of sleep health.
